# Real-Time Dual-Probe Monitoring and Correlation of FBRM and PVM Measurements during PUF Microcapsule Formation

**DOI:** 10.1021/acsomega.5c11155

**Published:** 2026-01-19

**Authors:** Başak Özeroğlu, Necati Özkan

**Affiliations:** † Department of Polymer Science and Technology, Middle East Technical University, Ankara 06800, Türkiye; ‡ Department of Mechanical and Composite Systems, Roketsan Inc, Ankara 06780, Türkiye

## Abstract

Microencapsulation enables the protection and controlled release of reactive liquids, however most characterization methods capture only endpoint properties. To address this gap, a real-time dual-probe approach using Focused Beam Reflectance Measurement (FBRM) and Particle Vision and Measurement (PVM) was employed to monitor the formation of poly­(urea–formaldehyde) (PUF) microcapsules containing dicyclopentadiene (DCPD) in this study. The four-stage synthesis was tracked *in situ* to evaluate emulsification, shell development and UF particle deposition under different stirring rates and poly­(ethylene-*alt*-maleic anhydride) (PEMA) concentrations. The method is applicable to capsule sizes measurable by both probes, i.e., > 10 μm for PVM and 1–1000 μm for FBRM. Within these ranges, the effective limitation in real-time monitoring arises from the PVM detection threshold. The results show that probe insertion introduces localized shear that influences capsule size distribution. A semiempirical power-law model was developed to reliably convert FBRM chord length data into true capsule diameters measured by PVM, using parameters derived from log-normal distribution fits. Overall, the integrated monitoring approach enhances understanding of process–structure relationships during microcapsule formation and provides a useful approach to improve microcapsule design in self-healing applications.

## Graphical abstract

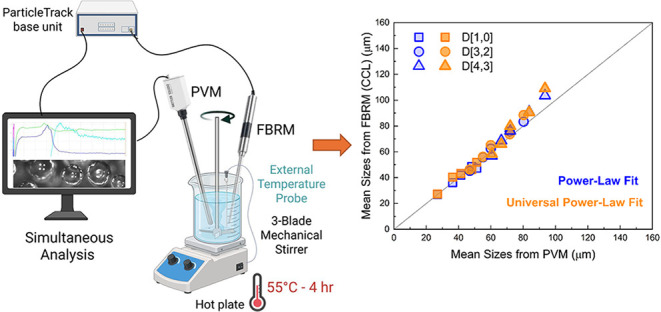

## Introduction

Microencapsulation is a widely used method for enclosing reactive or liquid materials within polymeric shells, forming discrete microcapsules that isolate and protect their contents. This technique enables controlled release and long-term stability across various fields, particularly in self-healing composites.^1^ By embedding microcapsules filled with healing agents into polymer matrices, materials can autonomously repair damage upon capsule rupture, which is a concept first demonstrated by White et al.^2^ and since extended to coatings,^3–14^ adhesives,^15^ electronics,^16^ biocompatible resins,^17,18^ insulation systems^19,20^ and fiber-reinforced composites.^1,21^

In these systems, microcapsules must meet certain requirements to work properly: they need to be stable over time, break open easily when damage occurs and mix well with the surrounding material without reducing its strength.^1,22^ These characteristics depend greatly on synthesis conditions, capsule morphology and dispersion quality.^23–26^ Microcapsules can be synthesized through two common *in situ* polymerization routes: the one-step method, in which shell formation occurs simultaneously with emulsification and the two-step method, where urea-formaldehyde (UF) prepolymers are first generated in solution and subsequently deposited onto dispersed droplets.^27^ The present work employs the one-step oil-in-water *in situ* polymerization approach, originally demonstrated for self-healing materials by Brown et al.^1^ and later refined and widely adopted in numerous studies^12–14,28–33^ on poly­(urea-formaldehyde) (PUF) microcapsule formation. These developments established the one-step route as the most commonly used synthesis method for self-healing systems, particularly due to its simplicity, reproducibility and suitability for producing well-defined PUF shells. Amino resins such as PUF are often chosen as shell materials because they provide strong, chemically resistant shells and good bonding with many polymer matrices.^24,34,35^

Most studies evaluate capsule size and morphology only after the synthesis is complete, typically using SEM or optical microscopy. However, these methods provide no information about how microcapsules form and evolve during the process, which limits real-time understanding of the formation mechanism.

To overcome these limitations, this study adopts a real-time dual-probe monitoring strategy involving Focused Beam Reflectance Measurement (FBRM) and Particle Vision and Measurement (PVM) to elucidate the microencapsulation mechanism of PUF-DCPD systems. This approach enables stage-wise tracking of microcapsule formation, agglomeration behavior and morphological transitions. While FBRM provides continuous chord length distributions, PVM offers real-time visualization of droplet, allowing synchronized interpretation of each synthesis stage (Stages A–D). Additionally, characteristic size descriptors, arithmetic mean (D­[1,0]), surface area moment (D­[3,2]) and volume moment (D­[4,3]), are monitored throughout the process to quantitatively support the visual findings and clarify structural evolution under varying synthesis conditions. The influence of synthesis parameters, such as PEMA concentration and stirring rate, as well as the potential hydrodynamic effects introduced by probe insertion, are also investigated in terms of their impact on microcapsule size and shell characteristics.

A key contribution of this study lies in establishing a statistically grounded correlation between chord length data obtained from FBRM and actual microcapsule sizes measured from PVM images. In the limited number of studies^36–38^ that utilize FBRM and PVM for microcapsule synthesis, FBRM has mainly been used for trend observation rather than quantitative analysis, while PVM is typically used only for image capture or visual inspection without being quantitatively analyzed or correlated with particle size measurements. Kutluay et al.^39^ demonstrated how the combined use of FBRM and PVM improved the understanding of crystallization by correlating optical data with morphological transitions. Earlier studies, those by Barrett and Glennon,^40,41^ focused on crystallization systems, while more application-driven work, by Greaves et al.^42^ and Zidan et al.,^37^ explored emulsion and microcapsule systems. In the few studies that attempted particle size correlations,^43^ empirical methods were used, but the results remained within ± 50% uncertainty bounds around a linear trend. Therefore, this study aims to develop a statistically robust empirical model that quantitatively links chord length measurements to actual capsule sizes, enabling reliable conversion of FBRM data into true microcapsule dimensions.

## Materials and Experimental Methods

### Materials

In this study, dicyclopentadiene (DCPD) was used as the core material. Urea served as the primary precursor for poly­(urea-formaldehyde) (PUF) shell formation, while ammonium chloride acted as the acid catalyst and hardening agent to promote UF prepolymer condensation. Resorcinol was included as an electron-rich phenolic compound, it reacts with formaldehyde and forms additional cross-links with UF prepolymers, enhancing the mechanical integrity of the capsule wall. A poly­(ethylene-*alt*-maleic anhydride) (PEMA) copolymer (*M*_*w*_ = 100 000–500 000, powder) was used as the emulsifier to stabilize the oil-in-water emulsion. A 37% formaldehyde solution provided the aldehyde source for UF polymerization, and 1-octanol was employed as a defoaming agent to improve emulsification stability. All chemicals were purchased from Sigma-Aldrich (USA) and used as received.

### Methods

#### Synthesis of Microcapsules

The one-step *in situ* polymerization method to synthesize PUF microcapsules was adapted from Brown et al.^1^ with minor modifications, as schematically illustrated in Figure 1. A 1:1.9 molar ratio of formaldehyde to urea was used. Urea, resorcinol, ammonium chloride and aqueous PEMA solution were mixed in deionized water under stirring. The pH was adjusted to 3.50 using 10% NaOH. DCPD was added in a controlled, river-like flow, followed by 1-octanol and dropwise addition of formaldehyde. The mixture was heated to 55 °C and stirred for 4 h.

**Figure 1:**
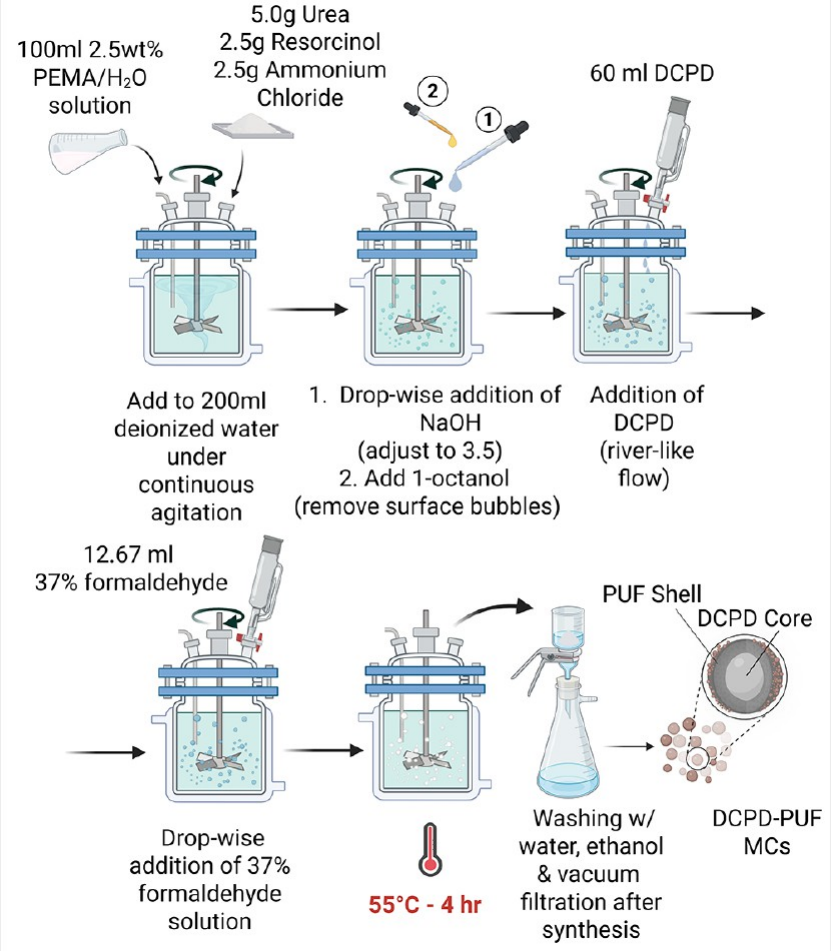
Schematic illustration of the *in situ* polymerization process used to synthesize DCPD-PUF microcapsules. *Created in BioRender. Ozeroglu, B. (2025) https://BioRender.com/ waejalx*

The same procedure was repeated under different conditions by varying the PEMA concentration and stirring rate, as shown in Table 1. The PEMA concentrations (1.5, 2.5 and 4 wt %) were selected to represent the established low, medium and high emulsifier levels reported for PEMA-stabilized oil-in-water microencapsulation. Concentrations below ∼1 wt % are known to produce unstable emulsions, whereas ∼1.5 wt % provides sufficient interfacial coverage for UF shell formation.^44–47^ The 2.5 wt % level, widely used in the literature,^28,48–53^ follows the standard formulation introduced by Brown et al.^1^ for PUF-DCPD microcapsules. Although higher PEMA concentrations can yield smaller and more stable droplets, studies also report practical limitations beyond ∼ 5 wt %, where excess emulsifier leads to overstabilization, increased viscosity and inhibited shell growth.^54^ Bolimowski et al.,^31^ for example, observed only a slight reduction in size above 5 wt % PEMA along with thinner UF walls and even the formation of UF nanoparticles rather than well-defined capsules. Therefore, 4 wt % was selected as the representative “high” level, approaching the upper effective limit where droplet size is minimized and shell formation remains robust.

**Table 1:** Experimental Conditions and Measurement Techniques Applied during Microcapsule Synthesis

PEMA Concentration (wt %)	Stirring Rate (rpm)	Measurement Technique
1.5	600	*In situ* FBRM and PVM^a^
2.5	400	*In situ* FBRM and PVM^a^
2.5	600	*In situ* FBRM and PVM^a^
2.5	800	*In situ* FBRM and PVM^a^
4.0	600	*In situ* FBRM and PVM^a^
1.5	400	OM and SEM (postreaction imaging)
1.5	500	OM and SEM (postreaction imaging)
1.5	600	OM and SEM (postreaction imaging)
2.5	400	OM and SEM (postreaction imaging)
2.5	500	OM and SEM (postreaction imaging)
2.5	600	OM and SEM (postreaction imaging)
4.0	400	OM and SEM (postreaction imaging)
4.0	500	OM and SEM (postreaction imaging)
4.0	600	OM and SEM (postreaction imaging)

a In *in situ*-monitored runs, probes were positioned at a 45° angle relative to the stirring direction and away from the vortex zone. Samples were also periodically collected at intermediate time points for SEM analysis.

For selected runs, *in situ* monitoring was performed using FBRM (ParticleTrack G600, Mettler Toledo, USA) and PVM (ParticleView V19, Mettler Toledo, USA) probes (Figure 2), which were positioned at a 45° angle relative to the stirring direction and placed away from the central vortex to minimize flow disturbance.

**Figure 2:**
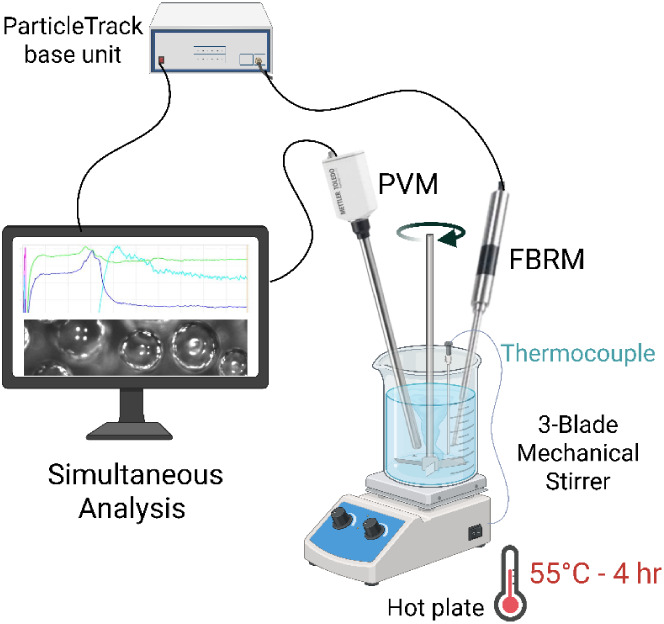
Experimental setup for *in situ* microcapsule synthesis with real-time monitoring using FBRM and PVM probes. *Created in BioRender. Ozeroglu, B. (2025) https://BioRender.com/ waejalx*

#### Characterization of Shell Morphology and Thickness

The surface morphology and shell structure of the microcapsules were examined using a Thermo Scientific Phenom XL G2 scanning electron microscope (SEM, Massachusetts, USA). To identify noncapsule residues or impurities, elemental mapping was performed via SEM-EDX analysis. Prior to imaging, sieved and air-dried microcapsules were mounted on conductive carbon tape and sputter-coated with gold to improve conductivity. In selected cases, capsule shells were intentionally ruptured using a spatula to expose the internal structure. For unruptured samples, cross-sections were prepared by embedding microcapsules in polydimethylsiloxane (PDMS) resin, curing and slicing with a razor blade. SEM imaging was used to assess shell thickness and integrity, with at least ten measurements taken per condition for statistical consistency.

#### Calculation of Various Mean Sizes

Capsule size measurements were carried out using optical microscopy (NIKON ECLIPSE LV100MD) images analyzed with ImageJ software (version 1.54g, USA). In order to obtain statistically robust mean size value, between 550 and 600 individual microcapsule diameters were manually measured for each sample.

The following general formula was used to calculate various mean particle sizes, arithmetic (D­[1,0]), surface area moment or Sauter (D­[3,2]) and volume moment or De Brouckere (D­[4,3]), based on corrected chord length (CCL) and PVM measurements, in order to assess the accuracy of the correlation models:^55^

1 $$D\left[m,n\right]={[\frac{\sum d_{i}^{m}N_{i}}{\sum d_{i}^{n}N_{i}}]}^{1/\left(m-n\right)}$$

where *N*_*i*_ is the count in size bin number *i* and *d*_*i*_ is the representative diameter of that size bin.

## Results and Discussion

### *In Situ* Process Monitoring of the Microencapsulation Process

Before microencapsulation started, the FBRM and PVM probes were placed into the reaction beaker after mixing aqueous PEMA, urea, ammonium chloride and resorcinol for 1 h under stirring. This step ensures that the PEMA surfactant is fully hydrolyzed and that the other chemicals dissolve properly. If hydrolysis is incomplete, residual polymer fragments may remain in the system. These can interfere with particle detection during microencapsulation by overlapping with UF polymer particles. As a result, the probes may fail to accurately detect UF particles. Real-time monitoring started immediately after 1-octanol was added and just before DCPD was introduced in each synthesis run with different parameters. This setup made it possible to observe the early stages of emulsification and core dispersion under the same conditions each time.

The results in the following sections are organized by process stage (Stages A–D), with each stage supported by three types of data: a schematic showing the microcapsule formation mechanism, a representative PVM image and the corresponding FBRM chord length distribution. This stage-by-stage layout make it easier to compare how the process changes over time and how it responds to different stirring rates and PEMA concentrations. For each stage, cumulative number distributions were reported and D­[1,0], D­[3,2] and D­[4,3] values were calculated. Comparing these values across stages provides detailed insights into how the capsules evolve during synthesis.

As illustrated in Figure 3a1–d1, four representative synthesis stages (Stages A–D) were identified, each corresponding to important points during the emulsification and *in situ* microencapsulation process.

**Figure 3:**
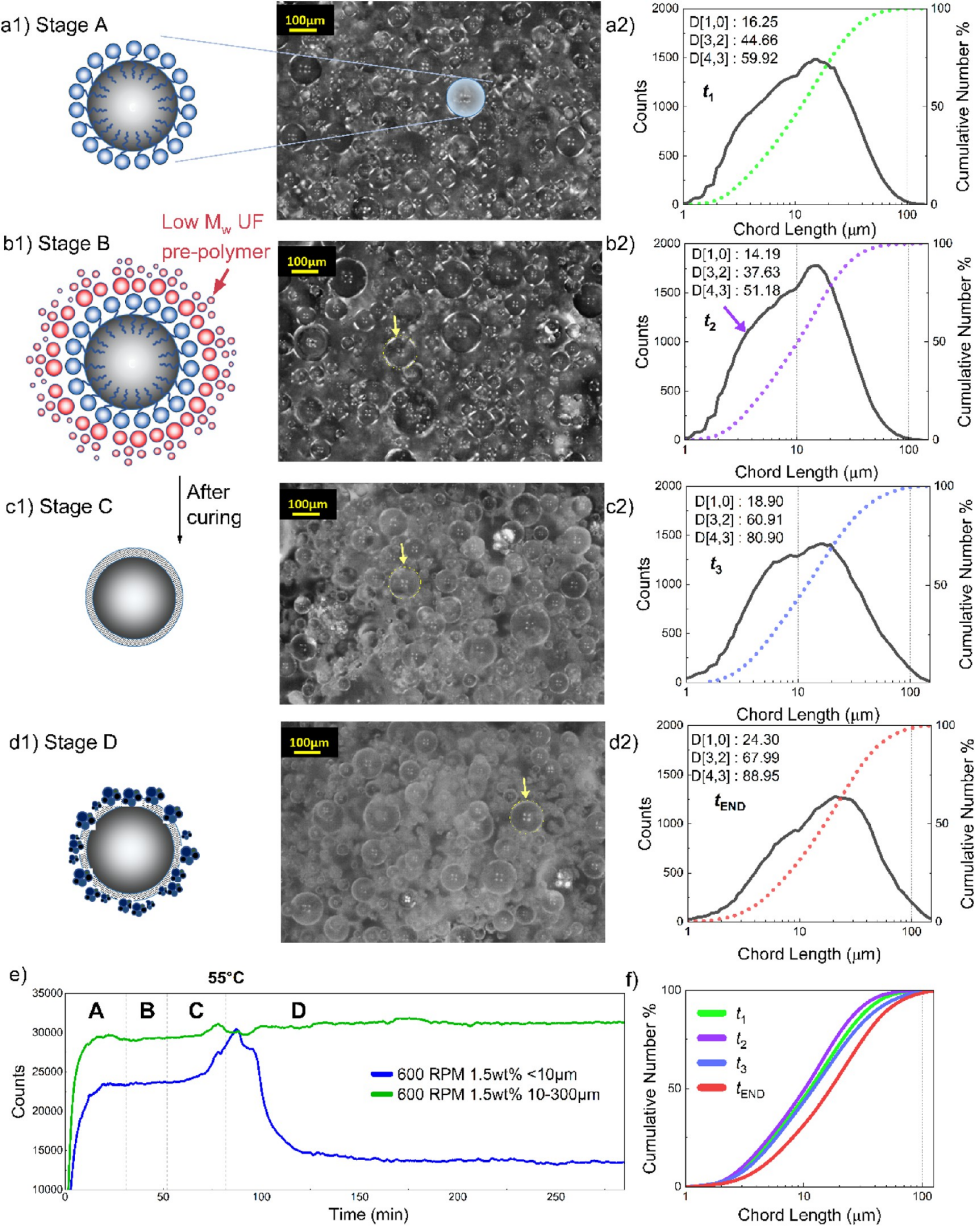
Real-time evolution of microcapsule formation monitored by PVM and FBRM across the four synthesis stages, showing schematic representations of droplet stabilization and shell development **(a1–d1)**, corresponding PVM images and chord length distributions **(a2–d2)** and particle count and cumulative chord-length trends during synthesis **(e–f)**.

Stage A involved the formation of a stable oil-in-water emulsion by dispersing DCPD into an aqueous phase containing urea, ammonium chloride, resorcinol and PEMA. Under constant stirring, a coarse emulsion developed and stabilized before polycondensation began.^46^ The stabilization period determined the droplet size and distribution from which microcapsules later formed. Suspension stability resulted from the balance between external shear and interparticle repulsion.^56^ Mechanical agitation fragmented the oil phase, while steric and electrostatic forces from adsorbed PEMA molecules prevented coalescence.^57^ Hydrolyzed PEMA, bearing carboxyl (−COOH) groups, adsorbed at the oil–water interface with its hydrophobic backbone toward the oil core and hydrophilic ends toward the aqueous phase.^47,58^ This orientation stabilized the emulsion and provided reactive sites for later UF polymerization.^59^ The count–time curve in Figure 3e shows an initial rise followed by a plateau, indicating a dynamic equilibrium after approximately 20 min. The PVM image (Figure 3a) revealed spherical droplets with clear boundaries, confirming emulsion stability at this stage.

Stage B began with the slow, dropwise addition of 37 wt % formaldehyde solution. Among Stages A–C, this stage exhibited the smallest mean particle size, with D­[4,3] decreasing temporarily to 51.18 μm (Figure 3b2, 3f), likely due to fine formaldehyde droplets dissolving rapidly in the acidic medium. The addition of formaldehyde initiated the formation of methylol-urea species in the continuous phase, while no major morphological changes were observed in the PVM images relative to Stage A.

Following formaldehyde addition, the temperature was gradually raised from 25 to 55 °C at 1 °C min^–1^, initiating acid-catalyzed condensation of methylol-urea intermediates.^26^ At this stage, PUF shell formation became dominant. A distinct pH drop from approximately 3.3–3.5 to around 2.0 for all PEMA concentrations indicated proton release during methylene-bridge formation, while linear UF prepolymers deposited at the oil–water interface, forming an initial shell around DCPD droplets. A smooth, compact wall developed as low-molecular-weight UF species adsorbed while still soluble; further condensation strengthened the layer into a rigid, continuous shell. Resorcinol promoted this process by reacting with formaldehyde and integrating into the UF network, improving mechanical integrity.^60,61^

A secondary population of UF particles formed via precipitation of high-molecular-weight prepolymers in the continuous phase. These particles aggregated and deposited on capsule surfaces, contributing to outer-shell roughness. FBRM results showed D­[4,3] increasing from 51.18 μm (Stage B) to 80.90 μm (Stage C) (Figure 3b2–c2), consistent with particle deposition. The PVM image at 55 °C displayed distinct capsule boundaries and a cloudy background, indicating the onset of shell solidification. These observations suggest simultaneous interfacial deposition and colloidal particle formation, characteristic of ongoing UF polycondensation.^1^

Stage D corresponded to the final hold period at 55 °C, enabling completion of cross-linking reactions. The mean capsule size increased to 88.95 μm (Figure 3d2), while PVM images showed opaque capsules with well-defined boundaries, indicating thicker, consolidated shells. This size increase was not due to coalescence, as droplets were already stabilized, but to continued UF particle deposition from the aqueous phase. Their accumulation on capsule surfaces further thickened the shell and enhanced surface roughness.

Postsynthesis SEM analysis confirmed numerous surface-deposited UF particles smaller than 10 μm (Figure 4). This agrees with the count–time data in Figure 3e, where sub-10 μm particles increased during Stage C and declined in Stage D as they attached to capsule surfaces. A slight rise in the 10–300 μm range (green curve) reflected capsule growth through colloidal UF deposition. Thereafter, the curve remained stable, consistent with a steady droplet population from Stage A. The cumulative distribution (Figure 3f) shifted toward larger sizes from Stage B to Stage D, confirming progressive UF particle deposition and agglomeration on capsule surfaces.

**Figure 4:**
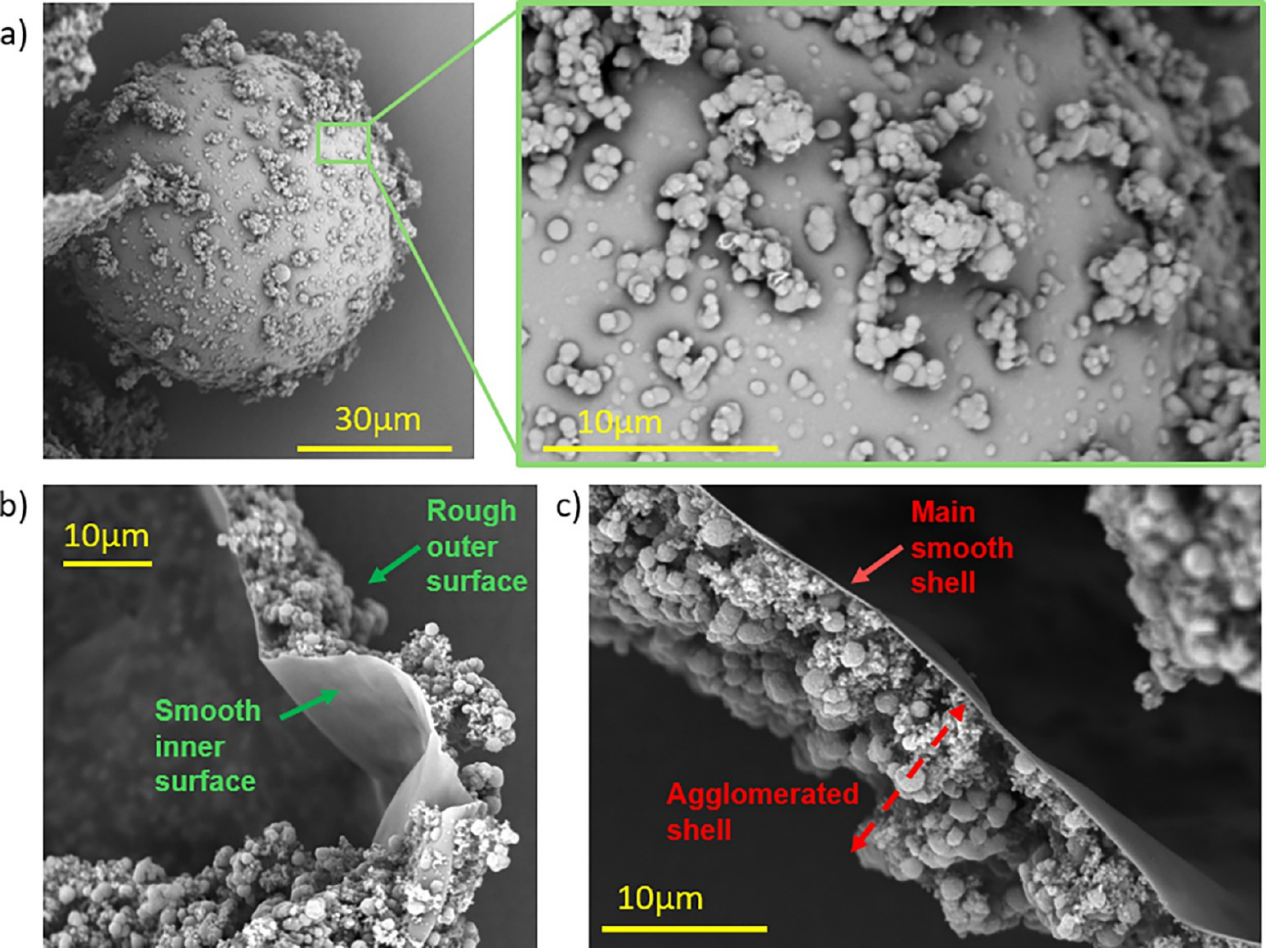
SEM images of PUF microcapsules. **(a)** Surface morphology and magnified view of the outer UF particle layer. **(b)** Cross-section showing the smooth inner membrane and rough outer surface. **(c)** Two-layer shell structure consisting of a smooth inner shell and an agglomerated outer layer.

These findings suggest that, whereas early shell formation is primarily governed by the deposition of soluble UF prepolymers at the oil–water interface, the later stages are dominated by ongoing polycondensation and the subsequent accumulation of UF particles on the capsule wall.

Figure 5 illustrates the effect of PEMA concentration on UF particle deposition. At 1.5 wt%, microcapsule surfaces are densely covered with UF particles, forming rough shells. At 2.5 wt%, coverage becomes more scattered, while at 4.0 wt%, capsules appear smoother and more uniform, indicating reduced deposition. This smoother morphology results from smaller capsule formation at higher PEMA content, which increases interfacial area for UF prepolymer adsorption. Consequently, fewer UF species remain in the aqueous phase for secondary polymerization, leading to less surface deposition. Additionally, the higher viscosity at elevated PEMA levels limits droplet movement during stirring, promoting finer emulsification.

**Figure 5:**
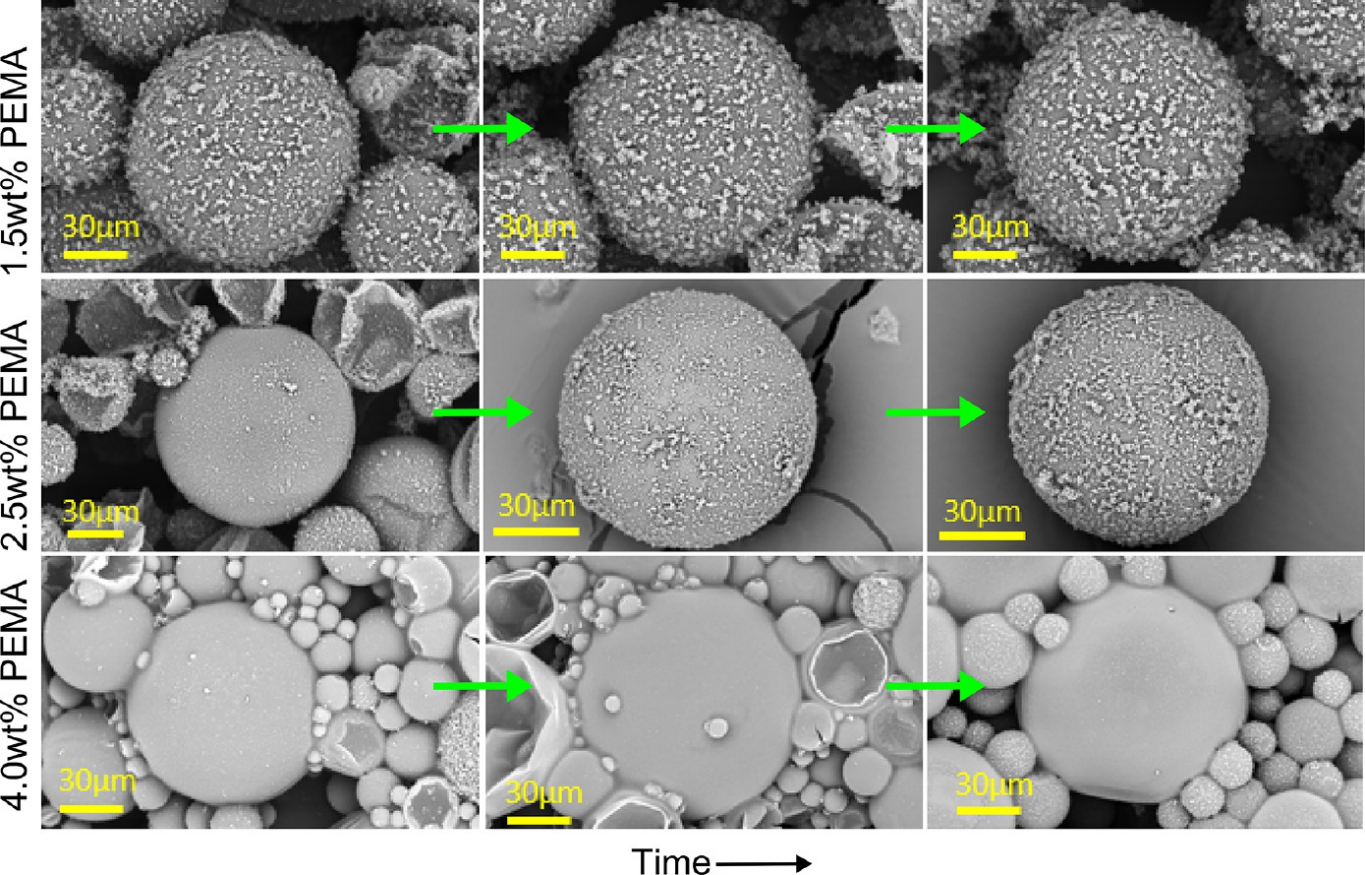
Effect of PEMA concentration on UF particle deposition and shell morphology. SEM images obtained at 1, 2.5 and 4 hours after reaching 55 °C show that lower PEMA concentrations lead to denser UF particle coverage, while higher PEMA yields smoother surfaces.

Overall, the real-time analysis revealed how shell formation evolves in distinct stages, shaped by both interfacial reactions and particle dynamics in the aqueous phase. The combined use of FBRM and PVM techniques enabled detailed *in situ* monitoring of synthesis medium to observe microencapsulation steps, providing valuable data for optimizing encapsulation procedures in future applications.

### Correlation between Chord Length and Capsule Size Using FBRM and PVM

One of the main challenges in comparing FBRM and PVM arises from how they measure particle size. FBRM records chord lengths (CL), line segments intersecting particles at random orientations, which are inherently smaller than the actual particle diameters observed in PVM due to geometric projection. FBRM measurements depend on the angle and location where the laser intersects the particle, the recorded CL values are often shorter than the actual particle diameter. This is illustrated in Figure 6, where the laser cuts through particles of various sizes at different points, producing CL values that do not reflect the full diameter unless the beam passes directly through the center.^41^ Therefore, a correction is needed to transform chord lengths into estimated microcapsule sizes, providing better consistency with image-based measurements.

**Figure 6:**
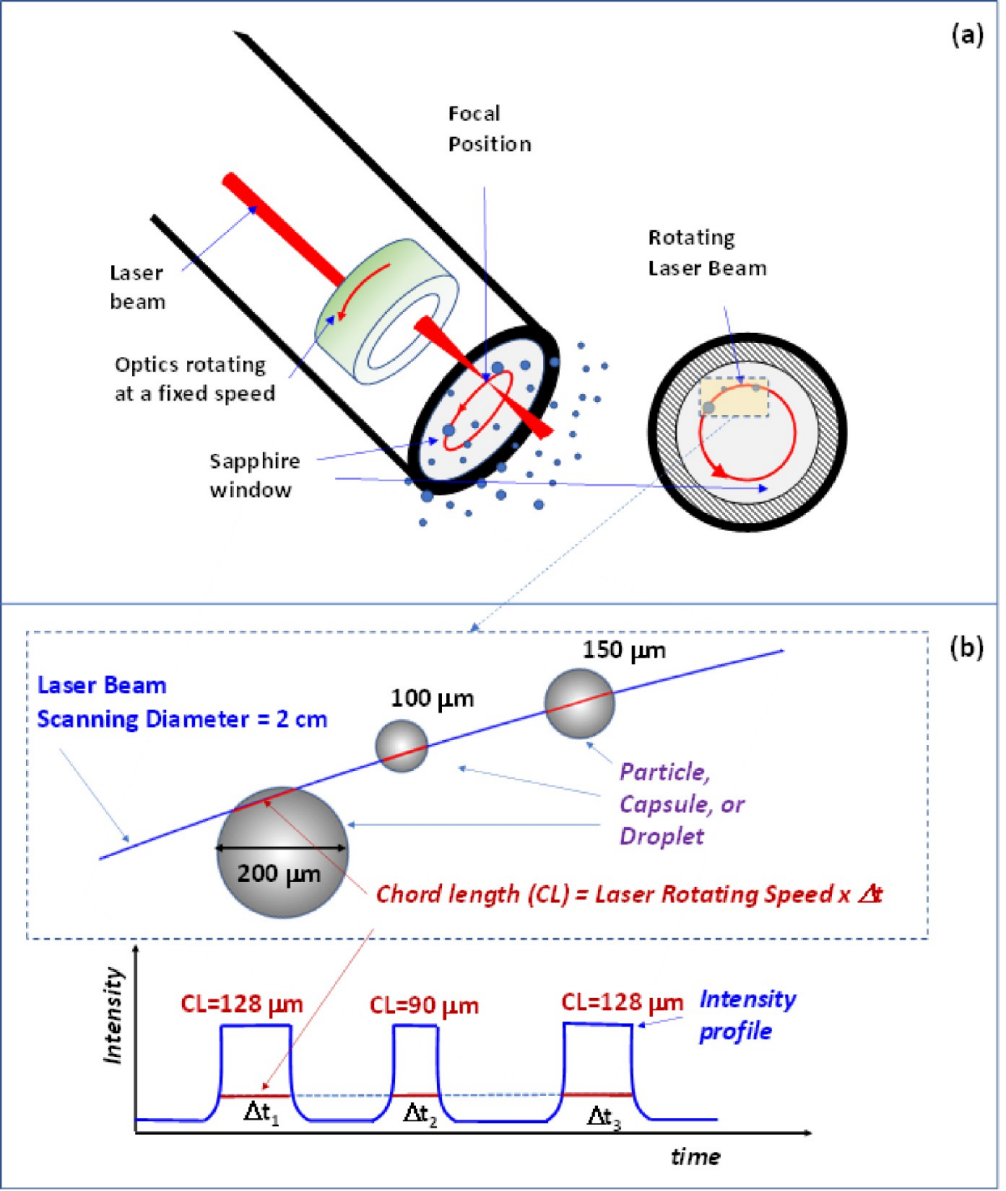
**(a)** Schematic illustration of how FBRM (*not to scale*) measures particle size by detecting CL as a rotating laser beam intersects particles along a circular scan path. Each interaction produces a signal and the signal duration corresponds to the length of the chord. **(b)** Definition of the chord length (*nearly drawn to scale*).

The particle size data from FBRM and PVM methods were first analyzed to enable correlation between datasets, ensuring statistical comparability across batches synthesized under different process parameters.

To evaluate whether the data followed a log-normal distribution, the cumulative undersize distribution curves for both chord length data (FBRM) and capsule size data (PVM) were constructed (Figure 7). From these, the cumulative undersize distribution curves for both data sets (PVM and FBRM), the geometric mean diameter (*D̅_g_*) and the geometric standard deviation (*σ_g_*) was calculated as follows:

2 $${\mathrm{D̅}}_{g}=D_{50}$$

3 $$\sigma_{g}=\frac{\sigma_{g1}+\sigma_{g2}}{2},{\;\sigma }_{g1}=\frac{D_{50}}{D_{15.87}},\;\sigma_{g2}=\frac{D_{84.13}}{D_{50}}$$

**Figure 7:**
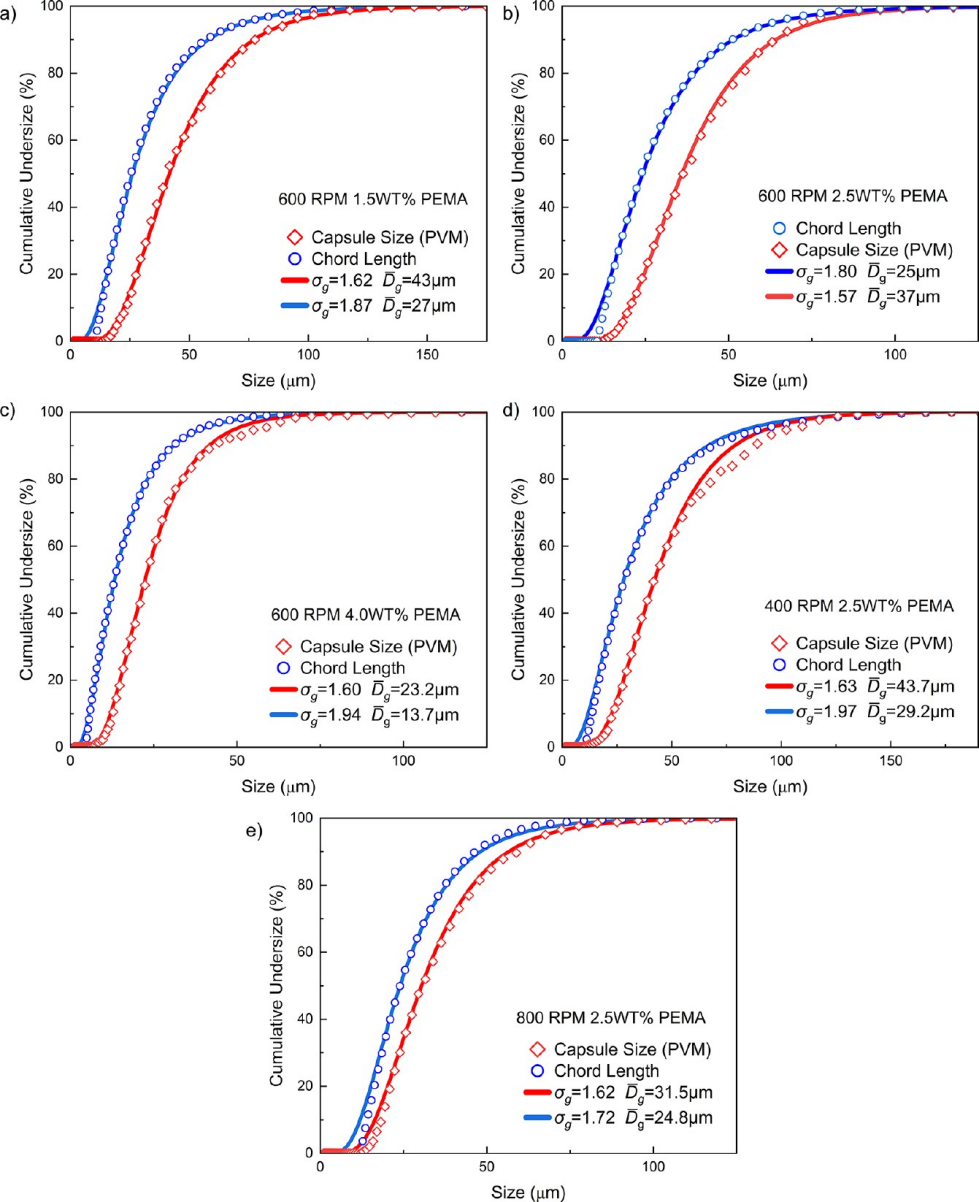
Cumulative undersize distribution curves for FBRM chord length data and PVM capsule size data under different synthesis conditions **(a–e)**. Experimental data points are shown with symbols and solid lines represent the corresponding log-normal fits based on *D̅*_*g*_ and *σ*_*g*_ values.

The chord length–count data were obtained from FBRM and the diameter values of capsules were measured manually from PVM images using ImageJ (about 550 capsules per condition). These parameters were then used to generate the log-normal size distribution using the following expression:^62^

4 $$f\left(D\right)=\frac{1}{\ln\left(\sigma_{g}\right)\cdot \sqrt{2\pi }}\cdot \exp\left[-\frac{{\left(\ln\left(D/{\mathrm{D̅}}_{g}\right)\right)}^{2}}{2\cdot {\left(\ln\left(\sigma_{g}\right)\right)}^{2}}\right]$$

To ensure a consistent comparison, the log-normal density distributions were calculated over the same bin widths used in the experimental data. These were then converted to cumulative undersize percentage distributions by numerical integration, allowing direct comparison with experimentally derived cumulative curves. As shown in Figure 7, all distributions, regardless of synthesis parameters, showed agreement with the fitted log-normal models, validating the assumption that both FBRM and PVM data follow log-normal behavior. Specifically, the log-normal probability plots in Figure 8 yield linear least-squares fits with coefficients of determination R^2^ ≥ 0.99 for both chord-length and capsule-size data sets, further confirming the statistical validity of the log-normal model.

**Figure 8:**
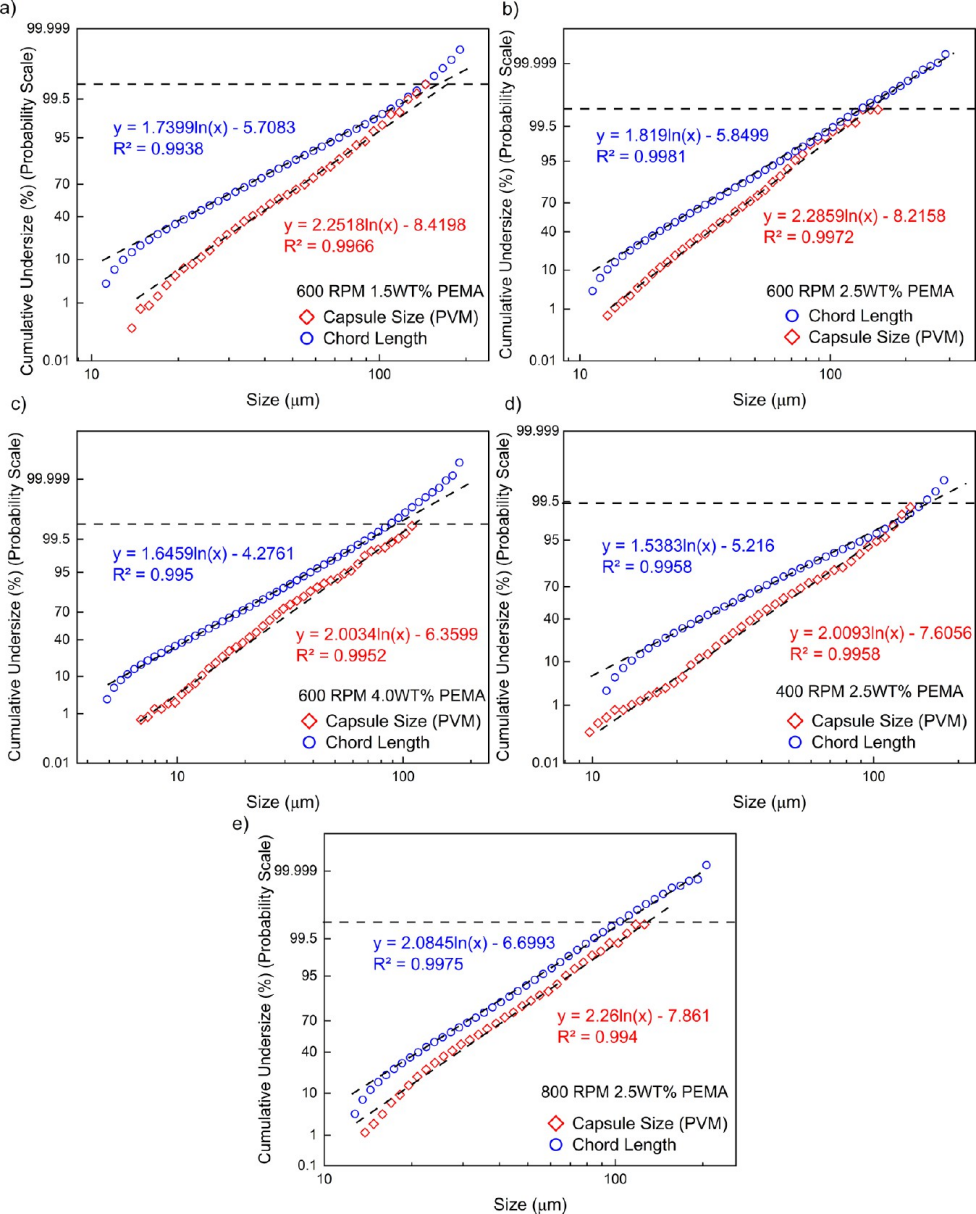
Log-normal probability plots for chord length (FBRM; blue) and capsule size (PVM; red) at five synthesis conditions: **(a)** 600 rpm / 1.5 wt % PEMA, **(b) **600 rpm / 2.5 wt %, **(c)** 600 rpm / 4.0 wt %, **(d)** 400 rpm / 2.5 wt % and **(e)** 800 rpm / 2.5 wt %. Linear least-squares fits (dashed) yield R^2^ ≥ 0.99, confirming log-normal behavior.

All data used in the optimization were carefully limited to the representative size range of actual microcapsules. Specifically, chord length values below 10 μm were excluded from data sets to eliminate the influence of small, noncapsule UF polymer particles, which form in the aqueous phase, with a portion of them depositing on the shell surface. These sub-10 μm particles were previously identified through SEM analysis (based on measurements taken from capsule surfaces shown in Figures 4 and 16), which showed that the particles agglomerated on capsule surfaces did not exceed 10 μm in diameter.

First of all, a correlation between microcapsule diameters obtained from PVM image analysis and chord length values measured by FBRM was performed through a one-parameter model using the following equation:

5 $$\mathrm{Capsule}\;{\mathrm{Size}}_{PVM}=B\cdot \mathrm{Chord}\;{\mathrm{Length}}_{\mathrm{FBRM}}$$

In this one-parameter model, the coefficient *B* operates as a proportionality factor that accounts for differences between the two measurement techniques. The value of *B* was selected in such a way that the corrected chord length geometric mean size (*D*_50(CCL)_) becomes equal to the geometric mean capsule size (*D*_50(PVM)_).

The *B* values calculated for the one-parameter model under each synthesis condition (i.e., varying RPM and PEMA content) are presented in Table 2. The corresponding cumulative undersize curves derived from the corrected chord length data fitted with this model are shown in Figure 9b. As seen from this figure, the one-parameter approach was not sufficient to accurately represent the full-size distribution.

**Figure 9:**
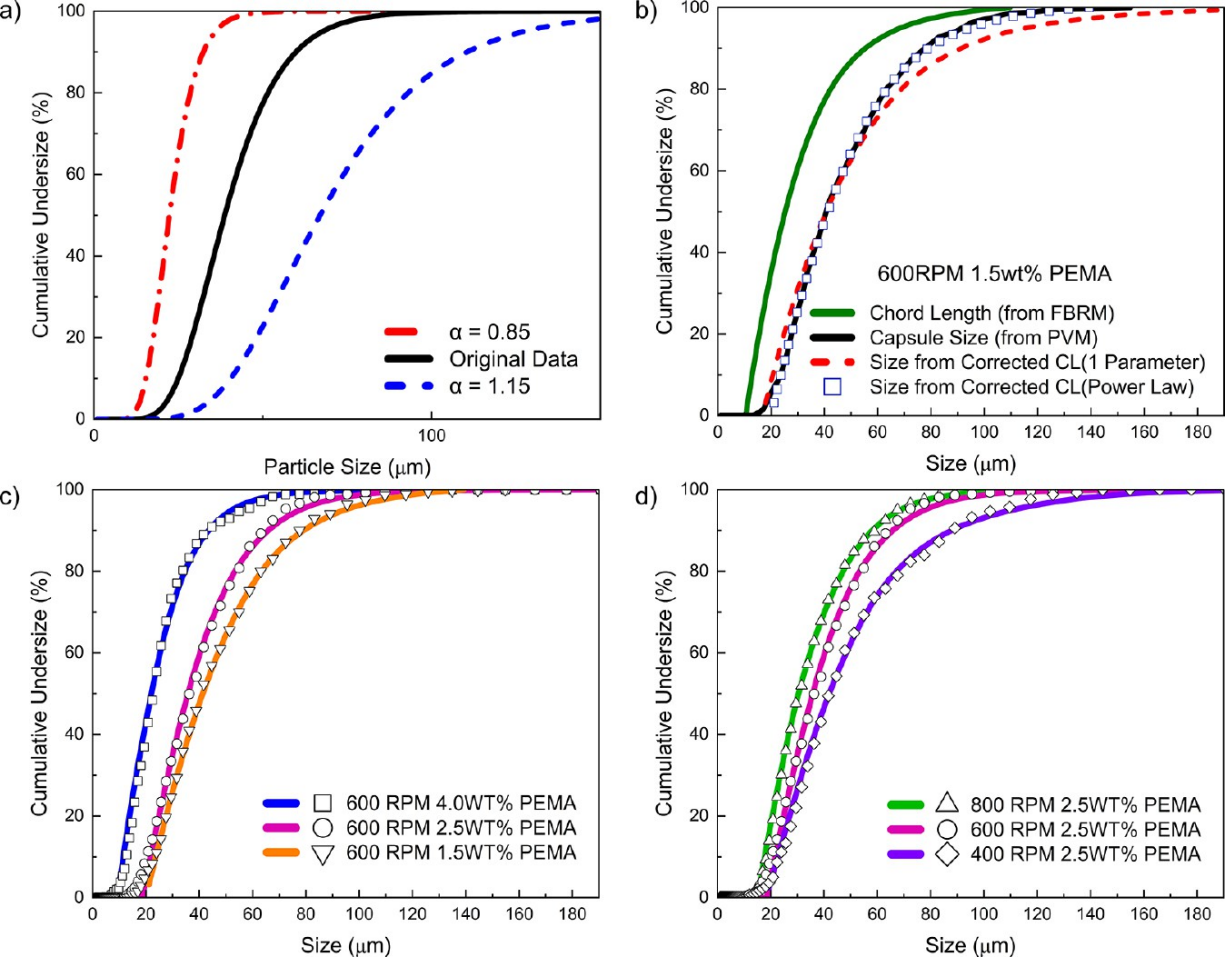
**(a)** Effect of the exponent *α*, broadening (*α* > 1) and narrowing (*α* < 1), on the geometric standard deviation (*σ*_*g*_). **(b)** Cumulative undersize distributions of FBRM CLs, PVM capsule sizes and size-corrected chord lengths (CCL) using single-parameter and power-law models (600 rpm, 1.5 wt % PEMA). **(c)** Model comparison at different PEMA concentrations. Symbols represent capsule sizes from PVM, while solid lines represent corrected chord length distributions from the power-law model.**(d)** Cumulative distributions at different stirring speeds for 2.5 wt % PEMA. The strong match between CCL and PVM data supports the reliability of the empirical power-law model.

**Table 2:** Particle Size Characteristics and Fitted Correction Parameters for Microcapsules Synthesized at Different PEMA Contents and Stirring Rates, Including the One-Parameter (*B*) and Power-Law (*A, α*) Models^a^

PEMA Content (wt %)	Stirring Rate (rpm)	Measurement Technique	D[1,0] (μm)	D[3,2] (μm)	D[4,3] (μm)	*D̅*_*g*_ (μm)	*σ*_*g*_	One-Parameter Correction Factor (*B*)	Power-Law Correction Factors (*A,α*)
1.5	600	CL (FBRM)	32.5	65.3	85.9	25.5	1.815	**1.5985**	**2.900**
		Capsule Size (PVM)	48.2	71.7	83.7	40.7	1.623		**0.816**
2.5	400	CL (FBRM)	37.7	82.3	106.2	28.5	1.907	**1.4739**	**2.590**
		Capsule Size (PVM)	51.2	80.3	93.5	42.1	1.710		**0.832**
	600	CL (FBRM)	29.8	55.8	75.2	23.9	1.748	**1.5230**	**2.990**
		Capsule Size (PVM)	41.4	60.0	72.0	36.4	1.582		**0.800**
	800	CL (FBRM)	27.2	45.0	57.1	22.4	1.681	**1.5990**	**2.910**
		Capsule Size (PVM)	36.3	55.1	66.4	30.7	1.592		**0.798**
4.0	600	CL (FBRM)	17.0	37.4	51.7	13.2	1.96	**1.7200**	**2.820**
		Capsule Size (PVM)	26.9	46.9	60.6	22.8	1.62		**0.797**
AVERAGE *(Universal Parameters)*								**1.5829**	**2.842**
									**0.809**

a Average values of *B, A* and *α* are also provided as universal parameters.

To create a more representative correlation between FBRM and PVM measurements, both the geometric mean and the distribution width should be considered. Therefore, relying only on one mean size value may lead to incomplete or misleading characterization. Reporting the full particle size distribution provides a more comprehensive and reliable description of microcapsule batches. For this purpose, an empirical correlation, which is a two-parameter power-law model, was proposed to accurately map the measured chord lengths onto the actual capsule size data:

6 $$\mathrm{Capsule}\;{\mathrm{Size}}_{PVM}=A\cdot {\left(\mathrm{Chord}\;{\mathrm{Length}}_{FBRM}\right)}^{\alpha }$$

In this expression, *A* serves as a size correction factor, while *a* adjusts for differences in the distribution profile (i.e., the geometric standard deviation) as illustrated in Figure 9a. In Figure 9a, the black solid line represents the log-normal distribution data with *D̅_g_* = 35 μm and *σ*_*g*_ = 1.40. If the alpha (*α*) value is larger than 1, the geometric standard deviation is increased to 1.48; however, if the alpha value is smaller than 1, the geometric standard deviation is decreased to 1.36. Therefore, the width of size variations between the chord length and the size from the PVM can be adjusted through the alpha index. This outcome suggests that the proposed power-law relationship qualifies as a semiempirical model, as it integrates both theoretically derived fitting principles and empirical validation across multiple synthesis conditions.

To determine the parameter values that yield the best fit, a two-step optimization approach was used. First, the value of *α* was estimated through an optimization procedure that minimizes the difference in distribution width between the two data sets. Specifically, the method of least-squares was performed over the geometric standard deviation values (*σ*_*g*_) obtained from the PVM measurements and the corrected chord lengths (CCL) fitted via the two-parameter power-law model (eq 6). The objective function used for this optimization is given by

7 $$\begin{array}{l} \min\\ \alpha \end{array}\left[{\left(\sigma_{g1,\mathrm{PVM}}-\sigma_{g1,\mathrm{CCL}}\right)}^{2}+{\left(\sigma_{g2,\mathrm{PVM}}-\sigma_{g2,\mathrm{CCL}}\right)}^{2}\right]$$

where the subscripts 1 and 2 correspond to the two forms of calculating *σ*_*g*_ as given in eq 3.This approach ensures that the selected *α* value aligns the distribution width between the two data sets.

After determining *α*, the parameter *A* was determined to align various cumulative size values (*D*_15.87_, *D*_50_ and *D*_84.13_) particularly focusing on representative percentiles of the distribution. In particle size analysis, especially when the data follows a log-normal distribution, the use of *D*_15.87_, *D*_18_ and *D*_84.13_ provides a statistically meaningful description of the size spread. Here, *D*_50_ corresponds to the median capsule size, while *D*_15.87_ and *D*_84.13_ represent the particle sizes at one geometric standard deviation (*σ*_*g*_) below and above the median in log-space, respectively. Unlike a normal distribution where these values are symmetrically distributed around the mean, in a log-normal system they become asymmetric in the linear scale: *D*_84.13_ is further away from the median than *D*_15.87_. This asymmetry reflects the right-skewed nature of the log-normal distribution commonly observed in microcapsule systems. Thus, these parameters allow not only the median size but also the geometric spread of the particle population to be quantified, providing a more representative characterization of capsule size distributions compared to relying solely on *D*_50_.

The objective function minimized via the least-squares minimization was:

8 $$\begin{array}{l} \min\\ A \end{array}\left[{\left(D_{15.87,\mathrm{PVM}}-D_{15.87,\mathrm{CCL}}\right)}^{2}+{\left(D_{50,\mathrm{PVM}}-D_{50,\mathrm{CCL}}\right)}^{2}+{\left(D_{84.13,,\mathrm{PVM}}-D_{84,13,\mathrm{CCL}}\right)}^{2}\right]$$

This two-step optimization approach was used to minimize differences in the geometric mean size (*D̅_g_*) and the geometric standard deviation (*σ_g_*) between the PVM measurements and the transformed FBRM data. It ensures a reliable correlation between the two data sets by correcting both the mean size and the distribution width. As seen in Figure 9b, the resulting values of *A* and *α* provided a strong agreement between the cumulative size distributions of the two methods, FBRM and PVM, across all synthesis conditions, as illustrated in Figure 9c-d. The improved fit was apparent in the alignment of both *D̅_g_* and *σ_g_*, which would not have been possible using the one-parameter model. This approach not only predicted various mean sizes but also the spread of the particle size distributions.

The resulting correction parameters, *B* for the one-parameter model and *A* and *α* for the power-law model, are listed in Table 2 for each synthesis condition. Their averages across all tests are reported as universal parameters, as they provide a generalized correction applicable to a wide range of synthesis studies. This aims to improve the model’s usability and make it applicable to a wider range of particulate systems.

As shown in Figure 10a, FBRM usually underestimates actual capsule diameters, particularly the arithmetic mean size (D­[1,0]). This observation aligns with earlier findings in the literature, which reported notable underestimation of particle size by FBRM in emulsion systems.^41,43^ In Figure 10b, when a one-parameter correction is applied, the agreement between corrected chord length values and PVM-measured diameters appears reasonable only for the arithmetic mean. However, this approach leads to significant overestimation in the surface-area-weighted (D­[3,2]) and volume-weighted (D­[4,3]) means. These results emphasize the limitations of the one-parameter model in accurately representing the full particle size distribution.

**Figure 10:**
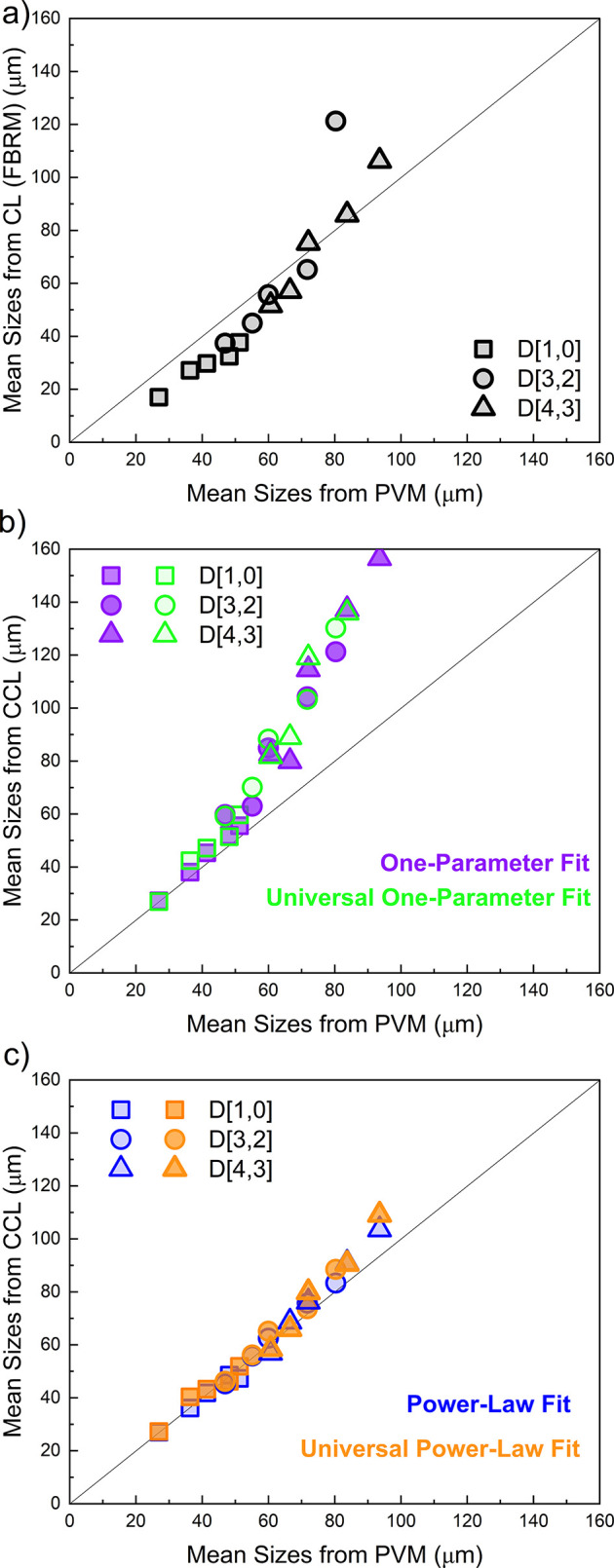
Comparison of mean particle sizes obtained from PVM and FBRM using three approaches: **(a)** uncorrected FBRM CL data, **(b)** one-parameter correction (condition-specific and universal) and **(c)** power-law correction (condition-specific and universal). *Solid line represents perfect matching*.

Moreover, this observation alone demonstrates that reporting only the arithmetic mean size is not sufficient for characterizing particle populations. While D­[1,0] may appear to provide acceptable agreement, it does not adequately represent the entire particle size distribution. Therefore, both D­[3,2] and D­[4,3] should be considered with D­[1,0] when reporting mean particle size and particle size distributions, in order to ensure a more accurate and representative description.

In contrast, the power-law model, which applies both a scaling factor *A* and an exponent *α*, provides a much better match across all three mean size values, as shown in Figure 10c. While the scaling factor *A* adjusts size differences, the exponent *α* corrects the width of the size distribution for improved alignment, leading to a closer match with the actual particle size values.

Notably, as seen from Figure 10, although power law provide a much better match across all three mean size values, there is still overestimation of D­[3,2] and D­[4,3]. This discrepancy becomes more apparent in the Cumulative Undersize (%) (Probability scale) vs Size plot shown in Figure 8. As highlighted by the black dashed line, a small number of chord length measurements (less than 0.5%) from the FBRM show a larger size than the capsule size from the PVM. Because of the nature of the chord length measurement principle, this should not happen. Although previous studies have reported that FBRM generally underestimates particle size compared to image-based techniques,^42,43^ in this study, overestimation was observed for the upper end of the size distribution, particularly above 99.5%. This deviation may arise from two possible causes: *(i) particle overlap in dense suspensions* or *(ii) the presence of large agglomerates or a combination of both* as illustrated in Figure 11. If high particle density is not the primary factor, the most likely explanation is the existence of large agglomerated particles (as seen in Figure 13c). These scenarios are consistent with findings reported in the literature, thereby supporting the reliability of this interpretation. In FBRM, the laser beam rarely intersects droplets exactly through their center, resulting in shorter measured chord lengths and thus underestimation of actual particle size^43^ is expected. However, as noted in earlier studies, overlapping droplets in concentrated emulsions increase the probability of detecting larger particle dimensions, as overlapping can cause multiple droplets to appear as one.^41,43^ This effect was especially pronounced in high-concentration systems, such as those containing 4 wt % PEMA, where PVM images clearly showed a denser microcapsule population compared to other samples. In such cases, overlapping or closely packed microcapsules are more likely to occur, causing the FBRM probe to detect merged chord events that correspond to artificially inflated size readings. Thus, the *α* exponent can be considered an effective means of offsetting the chord-length bias, producing size estimates aligned with the PVM measurements.

**Figure 11:**
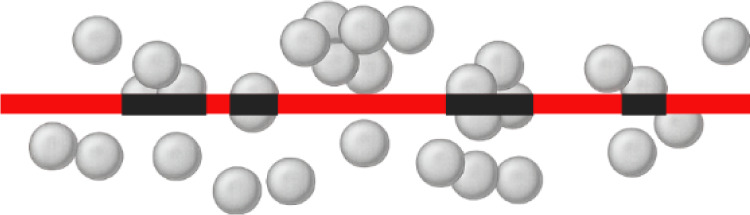
Schematic illustration of the FBRM measurement principle, showing the laser pathway (red) intersecting overlapped particles, agglomerates and the corresponding measured chord lengths (black). *Created in BioRender. Ozeroglu, B. (2025) https://BioRender.com/ waejalx*

Moreover, Schümann et al.^43^ noted that FBRM exhibits reduced accuracy when the particles are agglomerated (clumped). In experiments involving solid particles prone to agglomeration, the authors reported that FBRM “did not give reasonable output.” Such agglomeration can lead to an overestimation of the largest particle sizes in the distribution. This observation aligns with the results of the current system, where an overestimation becomes apparent at the upper end of the size range. As seen in the cumulative undersize (%) vs particle size plot in Figure 8, the number of large agglomerates appears to be less than 0.5%. Although these particles represent a small fraction of the population, their impact on D­[3,2] and D­[4,3] values is significant due to the nature of these metrics, which are strongly influenced by the surface area and volume of larger particles. The presence of large agglomerates is also supported by the SEM image shown in Figure 13.

The use of universal correction factors, calculated by averaging *A* and *α* values across all synthesis conditions, enabled the reliable prediction of mean sizes from corrected chord length data. As seen in Figure 10c, these results closely matched those obtained using condition-specific parameters, confirming the robustness of the universal model. Accordingly, the following expression is proposed as a generalized equation to estimate capsule size from FBRM data in polymeric microcapsule systems:

9 $$\mathrm{Capsule}\;{\mathrm{Size}}_{\mathrm{PVM}}=2.842\cdot {\left(\mathrm{Chord}\;{\mathrm{Length}}_{\mathrm{FBRM}}\right)}^{0.809}$$

To evaluate the statistical agreement between capsule size distributions obtained from corrected FBRM chord length data and those measured from PVM images, a two-sample *t-test* adapted for log-normal distributions was used. This analysis aimed to determine whether size distributions obtained from both methods could be regarded as statistically equivalent across different synthesis conditions. In this analysis, “Sample 1” refers to the power-law–corrected chord length, while “Sample 2” refers to the capsule size obtained from the PVM measurements.

The following equation was used for comparison, involving both the geometric means (*D̅*_*g1*_ and *D̅*_*g2*_) and standard deviations (*σ*_*g*_) of the two distributions:^100^

10 $$t=\left(\ln{\mathrm{D̅}}_{g1}-\ln{\mathrm{D̅}}_{g2}\right){\left(\left(\frac{\eta_{1}\eta_{2}}{\eta_{1}+\eta_{2}}\right)\frac{\nu }{\left(\eta_{1}-1\right){\left(ln\sigma_{g1}\right)}^{2}+\left(\eta_{2}-1\right){\left(ln\sigma_{g2}\right)}^{2}}\right)}^{1/2}$$

11 $$\nu =\eta_{1}+\eta_{2}-2$$

where η_1_ and η_2_ represent the number of observations in each data set, and *ν* is the degrees of freedom.

In all cases, the calculated *t*-values remained below the commonly accepted critical threshold of ± 2, indicating no statistically significant difference between the size distributions from PVM measurements and the corrected chord length distributions via both condition-specific and universal power-law fitting (Table 3). This confirms the effectiveness of the power-law fitting approach in aligning the two data sets under all synthesis conditions.

**Table 3:** Statistical Comparison between PVM Measurements and Power-Law–Corrected FBRM Chord Length Data, Showing *t-Test* Values for Both Condition-Specific and Universal Parameter Sets

		t-Test Value	
PEMA Content (wt %)	Rotation Speed (RPM)	Power-Law Fit (Condition-Specific)	Universal Power-Law Fit
1.5	600	1.471	1.780
2.5	400	0.035	0.860
	600	0.813	1.010
	800	0.274	0.350
4.0	600	0.960	0.320

The condition-specific model provided the lowest *t*-values, with the best agreement observed for the 2.5 wt % PEMA and 400 rpm condition, yielding a *t*-value of 0.035. The universal model showed slightly broader applicability, with *t*-values ranging from 0.320 to 1.780, indicating acceptable overall agreement.

These results show that while the condition-specific model offers the most precise alignment, the universal model still provides a reliable estimation of microcapsule sizes from the FBRM measurements. Therefore, the universal correction parameters can be confidently used for microcapsule characterization. As a result, the power-law model provides a consistent and reliable approach for comparing chord length and PVM data, particularly in microcapsule systems where particle size directly impacts end-product properties. The proposed correction approach improves how accurately *in situ* data reflects the actual size of polymeric microcapsules. The resulting semiempirical correlation (eq 9) enables straightforward conversion of chord length data into true microcapsule sizes. The *t-test* results confirmed that the corrected chord length values an agreement with the PVM measurements, even when using universal parameters. This supports the model’s reliability and its broad applicability under various synthesis conditions.

### Influence of Synthesis Parameters on Microcapsule Size and Morphology

To evaluate the effects of stirring rate and PEMA concentration on the morphology and size of PUF microcapsules, they were synthesized under varying conditions and analyzed using D­[3,2], optical microscopy (OM) and SEM. D­[3,2] was chosen for its sensitivity to surface area, providing a more accurate measure of droplet size changes.

Reliable evaluation of the effects can only be achieved after correct characterization has been carried out. Figure 12 highlights the necessity of sufficient particle counting to ensure reliable microcapsule size analysis. In many previous studies^48,63–66^ microcapsule size has been reported using only 50–250 measurements obtained from SEM or OM images. As shown, averaging fewer than 250 microcapsule measurements lead to considerable variation and deviation in mean size values. After approximately 450 measurements, D­[3,2] stabilizes with minimal change, confirming that a minimum of 450 microcapsules must be counted to obtain reliable size statistics. While Laser Diffraction technique (based on Mie Theory) has been widely used in previous studies,^57,67–71^ it has major limitations in distinguishing actual microcapsules from nonfunctional UF products.

**Figure 12:**
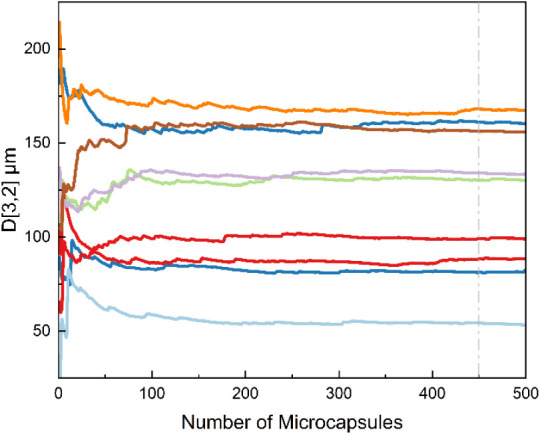
Cumulative D­[3,2] values as a function of the number of measured capsules under different synthesis conditions, with each curve representing an independent sample set. The gray dashed line marks the point where D­[3,2] becomes stable.

Figure 13a shows the laser diffraction particle size distribution curve of a representative microcapsule batch, showing two distinct particle populations. These populations were identified by peak deconvolution and represented in different colors, corresponding to distinct structures observed in SEM images (Figure 13b–d): spherical microcapsules, unreacted UF particles and noncapsular agglomerates. This observation highlights the limitation of the laser diffraction technique, as it does not yield a fully representative mean capsule size or accurate size distribution specifically for microcapsules, due to interference from secondary particulates and agglomerated structures.

**Figure 13:**
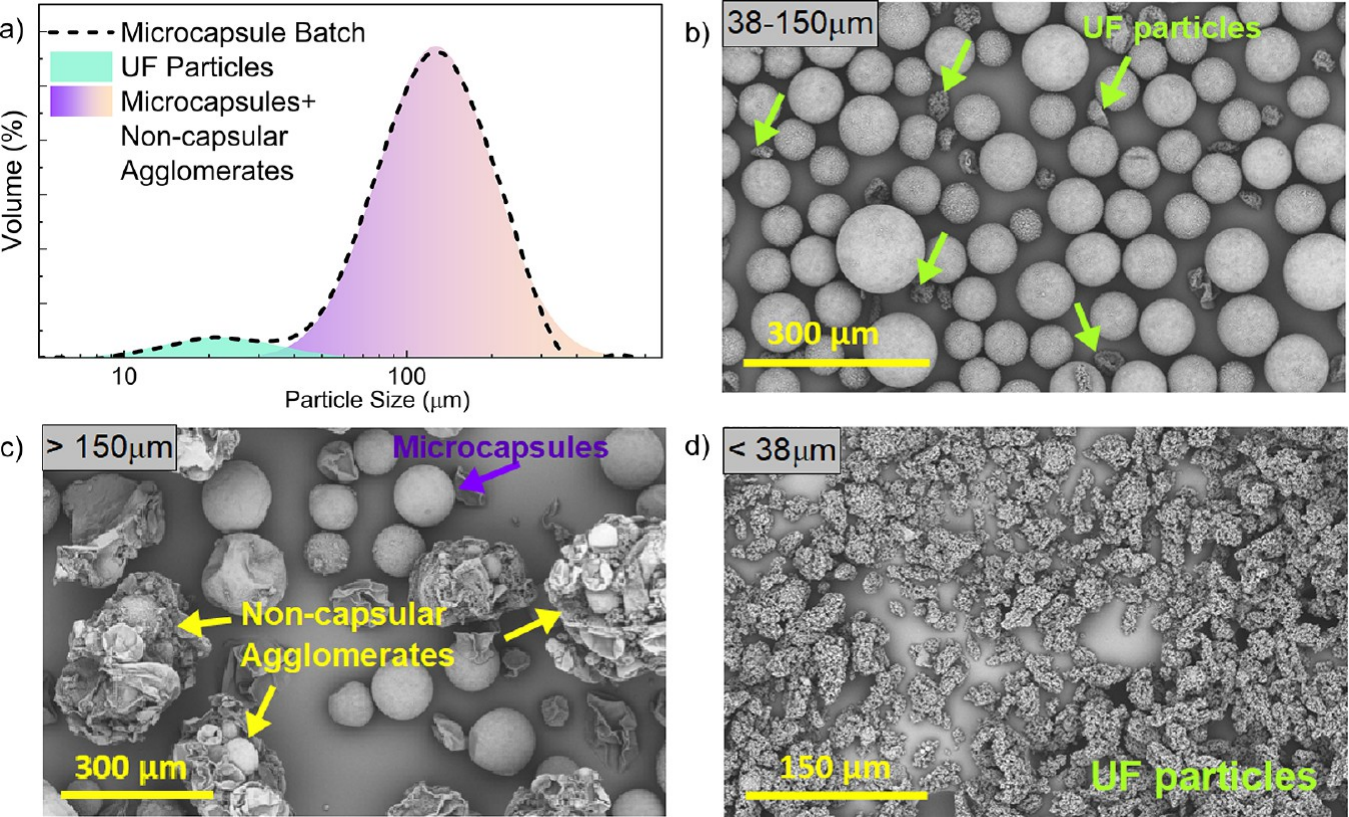
Volume-based particle size distribution for a representative microcapsule batch. **(a)** Full distribution. **(b-d)**: Separation of the main microcapsule population from smaller UF particles and noncapsular agglomerated species.

To further confirm the composition of these noncapsular residues, SEM-EDX analysis was performed (Figure 14). The elemental composition of the irregularly shaped particles revealed C, N and O contents consistent with poly­(urea-formaldehyde), similar to the microcapsules themselves.^13,14,72^ However, their morphological differences indicate that these UF structures were formed in the aqueous phase and did not contribute to shell formation. No foreign elements or byproducts were detected, confirming that these particles are not due to contamination or side reactions, but rather represent unutilized UF polymer that failed to assemble into the capsule wall during synthesis.

**Figure 14:**
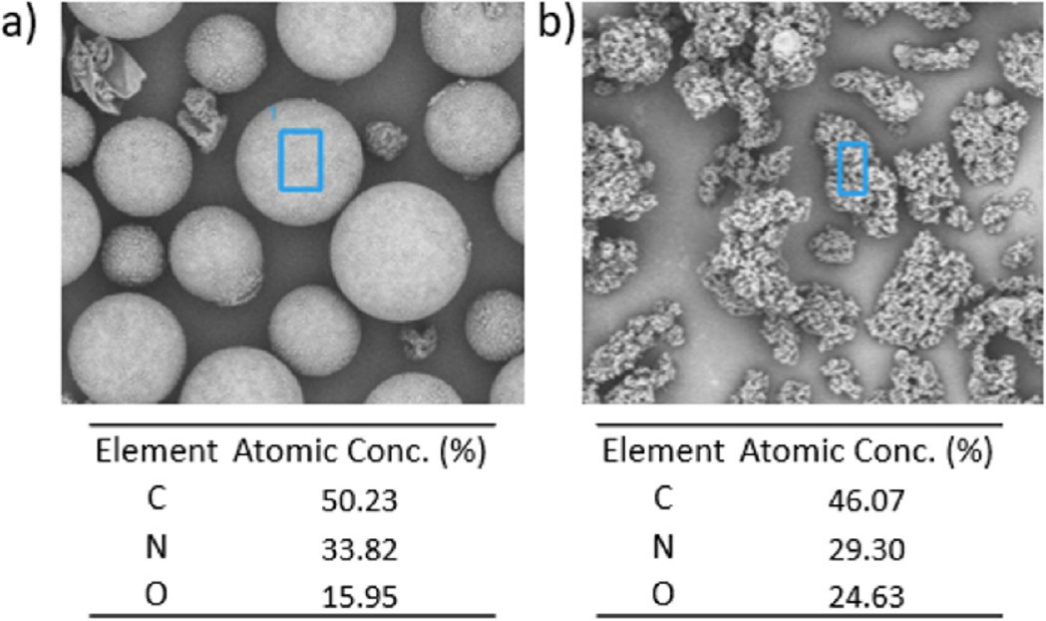
SEM–EDX analysis of noncapsule particles: **(a)** spherical PUF MCs and **(b)** agglomerated PUF particles observed outside the microcapsules. The elemental compositions confirm that these particles consist of PUF, indicating that they did not participate in shell formation. No additional byproducts or elemental impurities were detected.

For this reason, this study reports microcapsule size data based on direct imaging (OM and PVM), with a statistically robust sample size of at least 450 capsules per batch. This approach ensures accurate representation of true capsule dimensions and eliminates distortions caused by nonrepresentative measurements from laser diffraction measurements.

Figure 15a shows the variation of D­[3,2] with PEMA concentration at three stirring rates (400, 500 and 600 rpm) in the absence of probe interference. In all cases, microcapsule size decreased with increasing PEMA content. At 400 rpm, for instance, D­[3,2] dropped from ∼160 μm at 1.5 wt % to ∼120 μm at 4.0 wt %. Similar reductions occurred at 500 and 600 rpm. This trend results from enhanced interfacial stability at higher surfactant concentrations, which minimizes coalescence during emulsification and maintains smaller droplets throughout capsule formation. The effect is particularly evident at lower stirring rates, where mechanical shear alone cannot sustain emulsion stability. These results agree with previous reports,^1,58,73–76^ confirming that increased surfactant content reduces interfacial tension and produces finer, more stable droplets.

**Figure 15:**
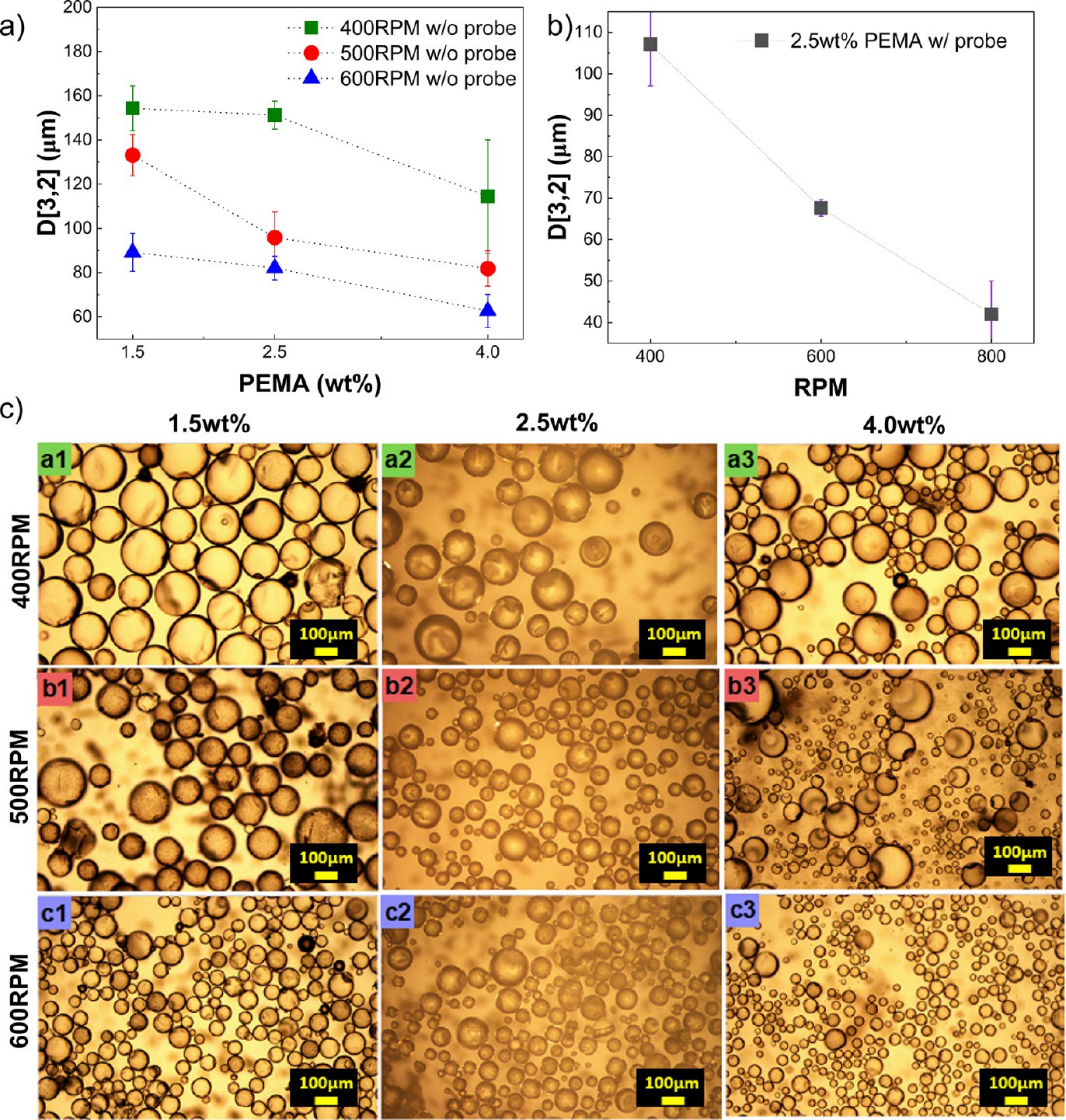
Effect of PEMA concentration and stirring speed on the size and morphology of microcapsules: **(a)** D­[3,2] as a function of PEMA concentration for samples synthesized without *in situ* probes at 400, 500 and 600 rpm. **(b)** Influence of stirring speed (400–800 rpm) on D­[3,2] values at a fixed PEMA content (2.5 wt %) when *in situ* probes were present. **(c)** OM images of microcapsules prepared under different PEMA concentrations and stirring rates (scale bar = 100 μm).

In Figure 15b, at a constant PEMA of 2.5 wt %, increasing stirring rate from 400 to 800 rpm reduces D­[3,2] from ∼110 μm to ∼43 μm, confirming that higher shear also yields finer emulsification. Figure 15c presents optical microscopy images showing how PEMA concentration and stirring speed affect microcapsule formation. Each row corresponds to a different stirring rate (400, 500, 600 RPM) and columns represent increasing PEMA concentrations (1.5, 2.5, 4.0 wt %).

Optical microscopy (Figure 15c) supports these observations: at 400 rpm & 1.5 wt % PEMA the capsules are bigger; increasing either PEMA or RPM produces smaller capsules, at 600 rpm & 4.0 wt % PEMA mainly small capsules are formed, though a few larger ones persist due to uneven shear fields. This nonuniformity arises from localized vortex gradients causing uneven droplet breakup, yet the overall effect of higher shear is the production of smaller microcapsules.

SEM and thickness analyses (Figure 16) showed a two-layer shell structure composed of a smooth inner membrane and a rough outer layer. The inner membrane thickness (160–260 nm) remained almost constant under all conditions, indicating that its formation is independent of stirring rate and PEMA concentration. This agrees with Brown et al.,^1^ who reported that the inner layer originates from interfacial deposition of low-molecular-weight UF prepolymers formed after formaldehyde addition. The rough outer layer forms later through the accumulation of colloidal UF particles from the aqueous phase.

**Figure 16:**
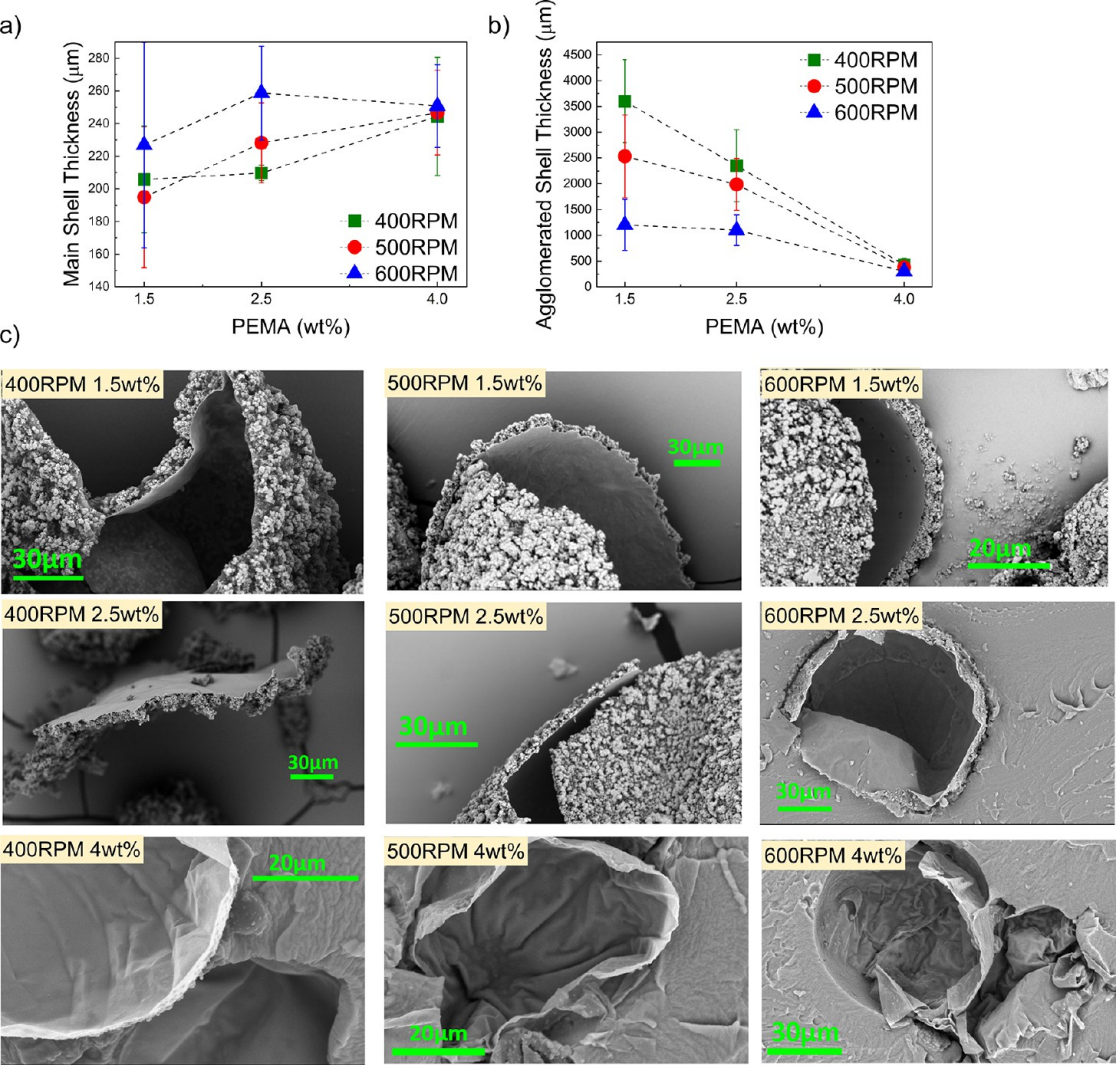
**(a)** Shell thickness trends for microcapsules synthesized at different stirring speeds and PEMA concentrations. **(b)** Agglomerated shell thickness values. **(c)** SEM images illustrating changes in shell morphology with varying RPM and PEMA content.

As shown in Figure 16b, the agglomerated outer layer became thinner with increasing PEMA content and stirring rate. At 400 rpm and 1.5 wt % PEMA, thicker shells were observed because limited interfacial area left excess UF prepolymers in the aqueous phase, which polymerized into free particles and later deposited on the surface. Despite this, the capsules kept a regular spherical shape, showing sufficient stabilization during emulsification. With increasing PEMA concentration, the agglomerated layer gradually decreased; at 4.0 wt %, the smoothest shell surfaces with minimal UF accumulation were observed (Figure 16c).

Higher stirring rates further reduced outer-layer thickness, as stronger shear limited UF clustering and improved droplet breakup, increasing the oil–water interface for UF deposition. Consequently, capsule surfaces appeared smoother at higher agitation levels. SEM images in Figure 16c confirm these trends, showing the combined influence of PEMA concentration and stirring rate on capsule morphology suitable for self-healing applications.

### Influence of Probe-Induced Hydrodynamics on Microcapsule Size Distribution

In this study, probe-induced hydrodynamic effects refer to local flow disturbances caused by the physical insertion of FBRM and PVM probes into the reaction medium. Although these probes are nonagitating elements, their presence generates regions of turbulent shear and velocity gradients near their surfaces. These localized effects can disturb emulsion stability, affect droplet breakup dynamics and consequently influence microcapsule size and morphology. Therefore, even without direct agitation, the mechanical interaction between the probe and the surrounding fluid is considered a non-negligible contributor to the microencapsulation environment.

Figure 17 shows that *in situ* FBRM and PVM probe insertion reduced surface-area-weighted mean diameter (D­[3,2]) under identical synthesis conditions. The localized turbulent shear generated around the probes enhanced droplet breakup and limited coalescence, especially at low stirring speeds. At 2.5 wt % PEMA and 400 rpm, D­[3,2] decreased from ∼ 155 μm to below 110 μm; similar reductions occurred at 600 rpm and 1.5 wt % PEMA. These results confirm that probe-induced shear improves local mixing and produces smaller microcapsules.

**Figure 17:**
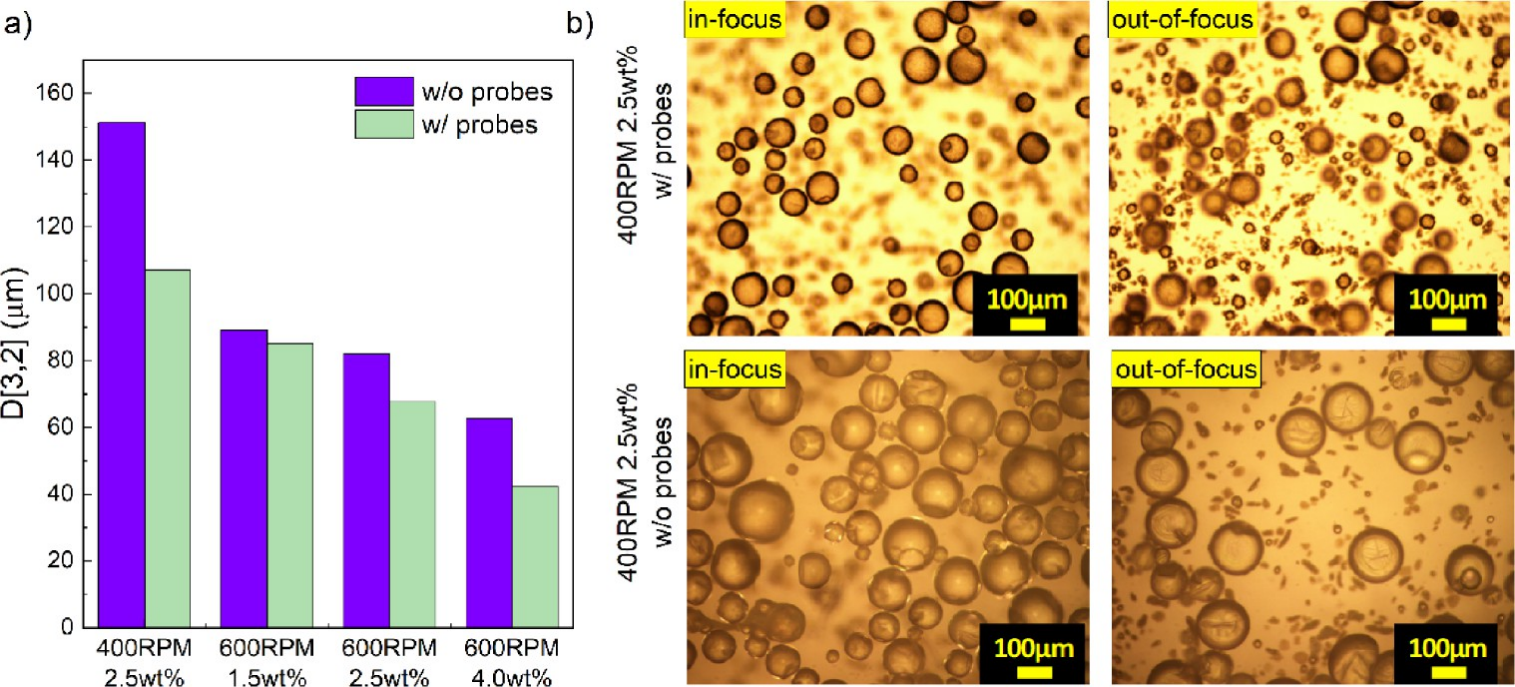
**(a)** D­[3,2] of microcapsules synthesized with (w/) and without (w/o) FBRM/PVM probe insertion under identical conditions. **(b)** Optical microscopy images of microcapsules produced at 400 rpm and 2.5 wt % PEMA: top row shows synthesis with probes and bottom row without probes, with in-focus images on the left and out-of-focus views of suspended UF particles on the right.

Figure 17b compares systems with and without probes. In-focus images show smaller capsules in the presence of probes, while out-of-focus images reveal higher concentration of free UF polymer particles in the aqueous phase. This suggests that the increased shear near the probes prevents some UF particles from attaching to the shell surface, leaving them suspended.^1^

These results show that although FBRM and PVM probes are designed for real-time monitoring and characterization, their insertion can significantly change the synthesis environment and affect the resulting microcapsule properties. This raises important concerns regarding the use of FBRM and PVM during synthesis, as their presence can introduce unintended shear effects that influence particle formation. As a result, particle size data obtained under probe-assisted conditions may not represent outcomes from conventional, probe-free synthesis. This issue is not limited to microcapsule systems but is broadly relevant to any study employing *in situ* probes. This highlights the need for careful evaluation of probe-induced hydrodynamics when interpreting or comparing data across synthesis studies.

## Conclusions

This study presents a comprehensive real-time analysis of PUF microcapsule synthesis using a dual-probe approach combining FBRM and PVM. By systematically varying stirring rate and PEMA concentration, their effects on emulsion stability, shell development and overall capsule morphology were quantified through time-resolved particle size data and *in situ* image-based morphological analysis.

The dual-probe setup enabled stage-wise monitoring of emulsification and shell formation, revealing that both synthesis parameters and probe-induced hydrodynamics significantly influence microcapsule size and structure. Although FBRM and PVM are nonagitating, their presence introduced localized shear, affecting droplet stability and UF particle dispersion. These findings highlight the need to account for such effects in *in situ* probe-based studies.

A major outcome of this study is the development of a semiempirical, two-parameter power-law model that enables the transformation of chord length measurements into accurate capsule size predictions. This correlation was based on the observation that both FBRM and PVM data sets consistently exhibited log-normal size distributions under all synthesis conditions. The log-normal behavior provided a robust statistical basis for establishing and validating the model. Importantly, the model was developed to be adaptable across different synthesis conditions, it was optimized using both condition-specific parameters for maximum accuracy and universal parameters to support broader applicability across varying synthesis conditions. *t-test* results and cumulative distribution comparisons confirmed the close agreement between the model-corrected FBRM values and PVM-measured capsule diameters.

While the condition-specific fits showed the closest alignment, the universal model still provided reliable size predictions, making it a practical and scalable tool for real-time particle size estimation in dynamic encapsulation environments.

Moreover, this study emphasizes the need for accurate and representative measurement practices. It was shown that measuring fewer than 450 microcapsules leads to significant variation in reported mean size, while laser diffraction techniques tend to overestimate capsule size due to the presence of noncapsular UF residues.

Overall, this study introduces a robust approach for real-time process monitoring and enhances the understanding of structure–property relationships in microcapsule formation and further supports the relevance of these microcapsules for self-healing applications, consistent with our previous work.^77^ The proposed universal power-law model may be extended to other particulate systems and future studies could explore its applicability beyond microencapsulation.
